# Development of a mobile application tracking stats and goals of young basketball players: design-based research

**DOI:** 10.3389/fpsyg.2026.1922244

**Published:** 2026-08-05

**Authors:** Ramazan Tascioglu, Canan Turgut, Ilker Usta, Serdar Kocaeksi

**Affiliations:** 1 Department of Physical Education and Sports, Faculty of Sports Sciences, Ardahan University, Ardahan, Türkiye; 2 Physiology of Exercise Laboratory Research Group, Department of Health and Human Performance, Faculty of Physical Activity and Sport Science, Universidad Politécnica de Madrid, Madrid, Spain; 3 Department of Physical Education and Sport, Graduate School of Health Sciences, Anadolu University, Eskisehir, Türkiye; 4 Department of Exercise and Sport Science, School of Physical Education and Sport, Istanbul Nisantasi University, Istanbul, Türkiye; 5 Department of Open and Distance Learning, Open Education Faculty, Anadolu University, Eskisehir, Türkiye; 6 Department of Physical Education and Sports, Faculty of Sport Science, Eskisehir Technical University, Eskisehir, Türkiye

**Keywords:** design-based research, goal setting theory, mobile application, norm development, performance feedback, social comparison theory, young basketball players

## Abstract

**Introduction:**

Mobile applications have emerged as a critical tool for supporting athlete development across various competitive domains. However, while numerous digital platforms exist for physiological monitoring, relatively few are grounded in goal-setting and social comparison theories to facilitate performance feedback and psychological self-regulation in youth team sports through an empirical norm-development process. Addressing this gap, this design-based research aimed to design, develop, and evaluate a mobile application to track the stats and goals of young basketball players.

**Methods:**

The research employed a design-based research methodology comprising planning, development, and improvement phases. During the planning phase, theoretical foundations, user characteristics, basketball performance norms, existing applications, and stakeholder expectations were examined to establish the design requirements. A user-centered mobile application was subsequently developed in collaboration with basketball players, coaches, software developers, and domain experts. Finally, players' experiences and evaluations were collected to improve the application and to explain how the design principles supported user engagement and performance development.

**Results:**

The norm development analysis of youth players (*n* = 1,556) demonstrated that age-stratified Z-scores and percentile distributions (1–99%), were used to develop a normative matrix, which was structurally integrated into the application across five core performance-profile badges (Rotation Players, Average Players, Stars, All-Stars, and MVP Candidates) and a National Player Candidates badge. The application's structural framework suggests that integrating objective performance statistics with individualized goal setting, social comparison, and continuous feedback offers a conceptually viable model that could support athletes' motivation, self-monitoring, and performance regulation. The iterative design process resulted in the mobile app Stats & Goals, which was found to help players track match statistics, compare their performance with peers, and set individual performance goals. Furthermore, a protocol was recommended for the development of similar applications.

**Conclusion:**

The study contributes to sport and exercise psychology by presenting an evidence-informed digital intervention grounded in established motivational theories while also providing a design framework for future sport technologies developed through design-based research. The proposed application offers practical implications for athletes, coaches, clubs, and researchers seeking to integrate psychological principles into digital performance support systems for youth sport.

## 1 Introduction

The rapid development of digital technologies has transformed how individuals access information (Dutton, 2004), communicate (Darwish and Lakhtaria, 2011), learn (Correa, 2008), and monitor their daily activities (Rainie and Wellman, 2019). This transformation has been particularly evident among adolescents and young adults, who are considered digital natives because they have grown up using digital technologies in their everyday lives (Goodyear, 2024). According to the Digital 2023: Türkiye report, smartphone ownership among individuals aged 16–64 reached 97.6%, while internet use and social media participation continued to increase across all age groups (Kemp, 2022, 2023). Among the primary reasons for internet use are searching for information, keeping up with current events, maintaining social relationships, and educational purposes. These trends indicate that mobile technologies have become an integral part of young people's daily routines and create new opportunities for supporting learning and personal development.

The increasing integration of mobile technologies into everyday life has also influenced educational (Correa, 2008) and sports environments (Peart et al., 2019). Mobile learning environments are perceived by users as flexible, portable, engaging, and independent of time and place, characteristics that particularly appeal to digital-native learners (Özsari and Saykili, 2020). Moreover, adopting the web for software development offers several benefits, such as no installation costs, automatic updates with new features for all users, universal access from any Internet-connected device, and independence from clients' operating systems (Dogan et al., 2014). Consequently, mobile applications are increasingly adopted to support training, monitor progress, and facilitate continuous interaction with users, owing to their low cost and user-friendly nature compared with traditional training systems (Özsari and Saykili, 2020; Dogan et al., 2014). Similar opportunities exist in sport, where mobile applications enable athletes to access information immediately, monitor their performance continuously, and receive individualized feedback regardless of training or competition schedules (Peart et al., 2019). A recent study demonstrated that mobile tracking applications significantly improve adolescents' physical fitness and motivational outcomes in physical education settings (Moudettir et al., 2025), and another intervention study with competitive youth cyclists aged 14–16 showed that self-monitoring via mobile and online platforms positively improved all performance measures across athletes (Ayvazo and Naveh, 2024). Moreover, contemporary evidence from elite youth football in Germany indicates that coaches' willingness to adopt mobile tools for live tracking, video feedback, and performance monitoring remains shaped by social expectations, resource constraints, and device-specific challenges (Ingwersen and Habenstein, 2026). Importantly, another study examined the effect of an AI-based performance analysis system on the motivation and self-efficacy of young football players and found that, among inexperienced players, short-term increases in autonomous motivation and self-efficacy were associated with AI-supported performance analytics (Zhang, 2026). Together, these findings indicate that the benefits of mobile performance tracking observed in adjacent youth populations can plausibly extend to youth basketball, where similar self-regulatory demands exist.

In competitive sport, objective performance data have become increasingly available through digital performance analysis systems (Connell and Spencer, 2020). In basketball, official match statistics provide comprehensive information regarding athletes' technical and tactical performances (Fauzi et al., 2024). However, providing statistical information alone does not necessarily improve performance (Fauzi et al., 2024). For performance data to be meaningful, athletes must be able to interpret it, evaluate their current performance, establish realistic improvement targets, and monitor their progress over time. Accordingly, mobile applications designed for athletes should not only display performance statistics but also facilitate self-regulation by supporting psychological processes associated with performance improvement. In youth basketball training, this psychological support can be significantly enhanced through the behavioral design of gamification elements and a deliberate exploration of player types, ensuring that digital incentives align with the diverse motivational profiles of young athletes (Feng et al., 2023).

Goal-setting theory offers one of the most extensively validated frameworks for promoting behavioral change and performance enhancement in sport psychology (Locke and Latham, 2013). Goals direct attention toward relevant performance elements, mobilize effort, increase persistence, and encourage athletes to develop effective learning strategies. Supporting this theoretical perspective, a meta-analysis of the sport and exercise psychology literature concluded that goal setting is an effective intervention for producing positive behavioral change (Kyllo and Landers, 1995). Researchers have consistently emphasized that effective goal-setting programs should incorporate multiple goal types—including outcome, performance, and process goals—together with regular evaluation and feedback (Locke and Latham, 2013; Burton et al., 1998; Weinberg and Gould, 2010; Widmeyer and Ducharme, 1997). These recommendations suggest that goal setting should be considered a dynamic self-regulation process rather than merely the establishment of desired outcomes.

Performance feedback constitutes another essential component of successful goal-setting interventions. Feedback informs athletes about the extent to which their current performance corresponds to their goals and enables them to determine whether additional effort or alternative strategies are needed (Ashford and Tsui, 1991). (Locke and Latham 2013) argued that goals regulate performance considerably more effectively when accompanied by continuous feedback than when feedback is absent. Furthermore, positive performance feedback has been shown to enhance perceived competence and intrinsic motivation (Vallerand, 1983; Vallerand and Reid, 1984), whereas performance-based feedback can mitigate the detrimental motivational effects of unsuccessful competitive outcomes (Vansteenkiste and Deci, 2003). Rather than focusing exclusively on winning or losing, emphasizing objective performance indicators may help athletes maintain motivation by evaluating their performance relative to their own standards (Weinberg and Gould, 2010; McAuley and Tammen, 1989).

In addition to goal setting and feedback, social comparison is an important psychological process that influences athletes' motivation and self-evaluation. During athlete development, individuals naturally compare their performances with those of teammates and competitors to evaluate their abilities and determine their position within the team (Festinger, 1954). Such comparisons are particularly evident during the early stages of team development, when athletes seek to understand their roles and evaluate their competencies relative to other team members (Weinberg and Gould, 2010). When based on objective performance indicators rather than subjective judgments, social comparison may facilitate more realistic self-evaluation and contribute to athletes' performance development.

This natural tendency for social comparison is deeply intertwined with the broader processes of team development and group dynamics. According to the linear theory of team development, groups progress sequentially through four distinct stages: forming, storming, norming, and performing (Weinberg and Gould, 2010). During the initial *forming* stage, athletes engage heavily in social comparison—evaluating teammates' strengths, weaknesses, and performance metrics—to determine their fit and establish their specific identity within the group (“we-ness” vs. “I-ness”). As teams transition toward the *norming* and *performing* phases, effectiveness becomes heavily dependent on two structural characteristics: role clarity (understanding position-specific responsibilities) and team productivity norms (agreed-upon standards for performance and effort; Carron and Hausenblas, 1998; Munroe et al., 1999). Strategic interventions that focus on clarifying individual roles and reinforcing objective group norms have been shown to significantly enhance team cohesion and reduce social loafing (Kocaekşi, 2015; Martin et al., 2013). In youth team sports like basketball, where formal roles (e.g., point guard, center) carry distinct requirements, providing athletes with objective performance feedback and role recognition is crucial for fostering role acceptance (Beauchamp et al., 2005). Consequently, evaluating these metrics with scientifically validated, empirical data rather than subjective judgments helps teams navigate early-development conflicts more smoothly, thereby establishing solid productivity norms directly tied to performance outcomes (Kagan, 2015).

These theoretical perspectives indicate that effective digital interventions for athletes should integrate objective performance statistics, individualized goal setting, continuous performance feedback, and opportunities for social comparison within a single environment. Although numerous mobile applications are available for monitoring physical activity, fitness, and exercise behaviors (Peart et al., 2019), relatively few have been developed specifically to support the psychological aspects of development grounded in established motivational theories (Connell and Spencer, 2020; Kokoç and Hayit, 2020).

Because many apps focus on adult basketball (Fauzi et al., 2024), this limitation is particularly important in youth basketball. In Türkiye, the Basketball Youth League was established to support talented young players in their transition toward professional basketball by providing greater competitive opportunities (Tinaz et al., 2017). Although official match statistics are routinely collected and published by the Turkish Basketball Federation, these data are primarily presented in descriptive form and offer limited support for athletes' self-regulated performance development. Transforming these objective statistics into individualized performance norms, meaningful feedback, and goal-setting opportunities may provide a practical means of supporting athletes' motivation and long-term development while helping coaches monitor player progress.

A recommendation by (Durdubaş 2020) indicates that this research will help fill a gap in the literature. Durdubaş investigated the effect of a season-long multi-level goal-setting intervention on team variables in his dissertation. At the conclusion of his dissertation, Durdubaş proposes investigating the tracking of individual goals and the provision of performance-based feedback through mobile applications. It is expected that the app developed in this study could be used to answer this question. To address this need, this design-based research aimed to develop a mobile application for youth basketball players. The main research question guiding this research was: “How can a mobile app be developed to support youth players' efficiency?”

## 2 Materials and methods

### 2.1 Research design

**Figure 1:**
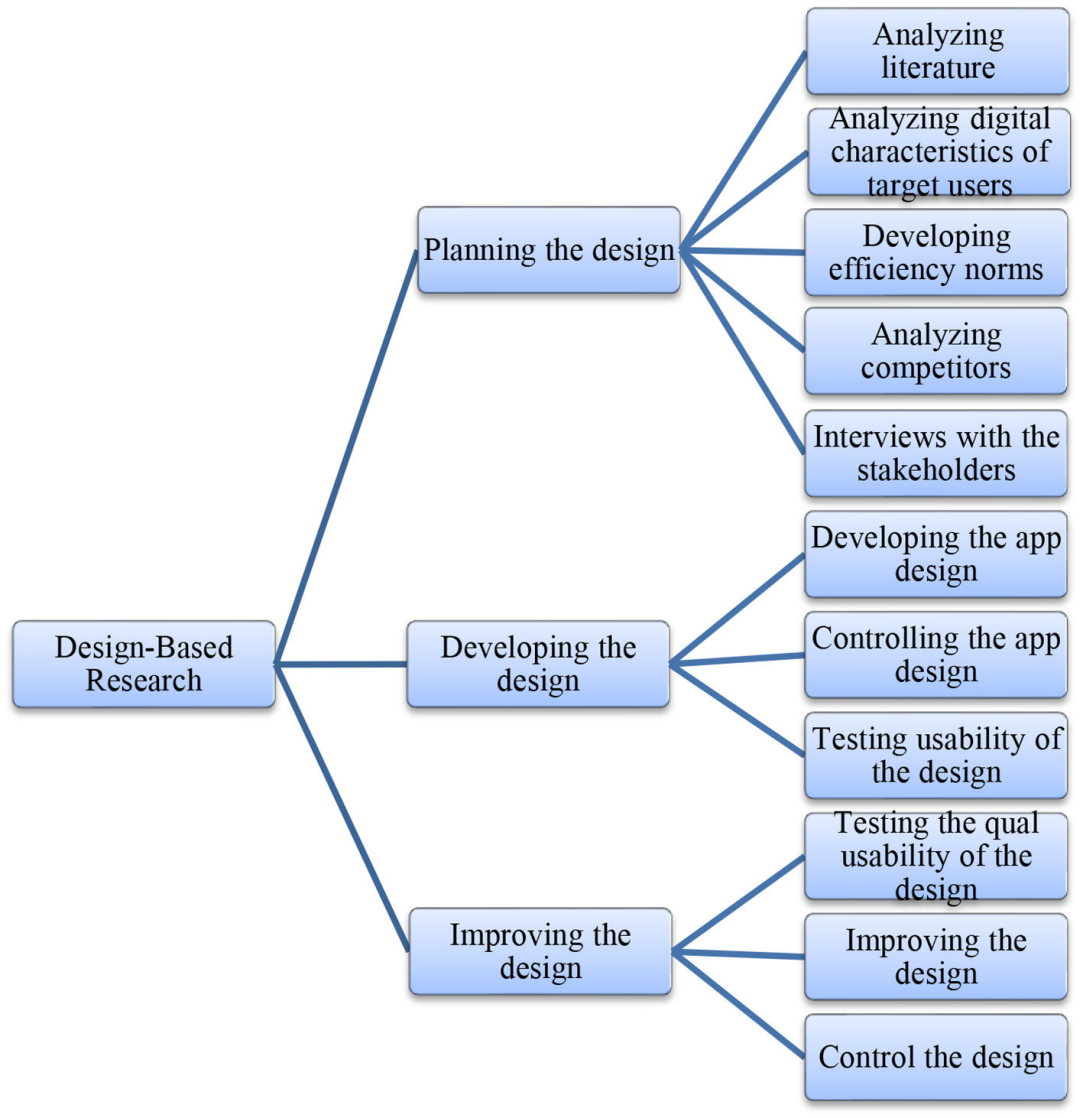
DBR design.

This research employed a design-based research (DBR) methodology to design, develop, and evaluate a mobile application that supports the monitoring of performance statistics and individualized goal setting among young basketball players. DBR is a contextual, grounded, integrative, interactive, and pragmatic methodology that aims to generate both practical solutions and theoretical knowledge through iterative cycles of design, implementation, evaluation, and improvement (Wang and Hannafin, 2005; Eric et al., 2003; Figure 1). Furthermore, each improvement cycle had 3 steps. These were the implementation, evaluation, and improvement steps. The first improvement step was linked to the second implementation step.

Since the purpose of this research is to develop a mobile application specifically tailored to young basketball players, user-centered design (UCD; Huang et al., 2019) was adopted to ensure that the end users directly influence how the digital artifact takes shape. Grounded in this approach, the development process was structured around the four key activities of UCD. First, the specific context of youth sports and basketball tracking was comprehensively understood and specified. Second, the unique requirements of young athletes were identified. Third, evidence-informed design solutions embedding motivational theories were generated. Finally, these designs were iteratively evaluated based on direct user feedback to refine the application's functionality. By systematically executing these four operational activities, the design process transformed raw psychological and performance principles into an intuitive, user-validated digital intervention.

In the planning phase, young basketball players were explicitly embedded not as active interviewees but directly through their performance data, utilizing their efficiency scores to develop the foundational age- and season-specific norm matrices (*n* = 1,556). This approach ensured that the application's core functionality was built on the youth players' on-court output. During the norm development process, coaches and former elite players acted as experts for validating norms. In the improvement phase, transitioning from “designing based on user data” to “refining directly with users” through qualitative usability evaluation. Thus, when examining all research processes, the final mobile application is directly shaped and validated by the youth athletes within the UCD framework.

In this user-centered design context, a user experience (UX) research methodology, including face-to-face and online moderated usability testing (Türk, 2023), was used to uncover problems in app design, identify improvements, and identify features to add (Cirucci and Pruchniewska, 2022).

DBR allows the use of multiple data collection methods. Both quantitative and qualitative data were used in this research. (Greene et al. 1989) categorized five general purposes of mixed-methods research: (i) triangulation, (ii) complementarity, (iii) development, (iv) initiation, and (v) expansion. In this research, the aim of using quantitative and qualitative data is to provide complementarity, development, and triangulation.

The research followed the guidelines of the Declaration of Helsinki and was approved by the ethics committee of Anadolu University (No: 68215917-050.99). In addition, the Turkish Basketball Federation gave written consent for the research (No: 2019/0939).

### 2.2 Participants and sampling methods

Consistent with the principles of design-based research, different stakeholder groups participated throughout the research, aligned with the objectives of each stage. Instead of recruiting a single group of participants, the study involved an interdisciplinary collaboration among end users, practitioners, technology developers, and domain experts to ensure that the mobile application addressed both practical requirements and theoretical design principles. Moreover, key stakeholders—including basketball coaches, former elite players, and sports science experts—actively contributed to the validity and reliability of the platform's features, bridging the gap between theoretical constructs and youth athletes' functional needs.

Because participant involvement varied across the iterative phases of the design-based research process, stakeholder contributions fluctuated in response to the objectives of each design activity. This collaborative structure enabled continuous validation of design decisions through multiple perspectives while ensuring that the resulting application reflected both theoretical foundations and practical user expectations.

In the planning phase, a full sample approach within a finite universe (Fraenkel et al., 2019) was used because data from all 1,556 players in the entire league universe across the five consecutive seasons were used to develop norms. In the improvement phase, maximum variation sampling (Teddlie and Tashakkori, 2009) was used to maximize the range of players' perspectives and obtain full diversity of players from each efficiency norm group. So, during the improvement phase, the qualitative usability (user) testing (Türk, 2023) was done with 5 players who were selected from the active and former players of different norms and different playing positions across the five consecutive seasons. Since the sample selected for the qualitative part of the research is a part of that used in the quantitative part, the sampling method in this mixed-methods research is nested (Collins et al., 2007). The participants in the research are as follows, according to the stages of DBR.

A total of 1,556 basketball players (Age M ± SD: 17.73 ± 0.89) participated in the planning phase to develop norms to be used as goals. The developer and designer collaborated with the researcher during the development and improvement of the application architecture and user interface. Five basketball players (Age M ± SD: 21.40 ± 1.52) participated in the improvement phase to identify user needs, test application prototypes, and provide feedback on usability and functionality (Table 1).

**Table 1:** Demographic and operational characteristics of participants across the three phases.

Phases	Seasons			*n*	Min.	Max.
Planning phase	2017–2018 2018–2019 2019–2020 2020–2021 2021–2022 Total			300	16	19
				303	15	19
				311	16	19
				304	16	20
				338	16	20
				1,556	15	20
Developing phase	**Codes**			**Gender**	**Education level**	**Expertise**
	FSD1 GD1			Male	Master of science	Full-stack developer
				Male	Master of science	Graphic designer
Improving phase	**Name**	**Season**	**Position**	**Age (years)**	**Seasons norms**	**Age norms**
	P1	2017–2018	SF	23	Stars	Stars
		2018–2019			Stars	Stars
	P2	2017–2018	G	23	Average player	Average Player
	P3	2019–2020	PF	21	Stars	Stars
	P4	2020–2021	C	20	All-stars	All-stars
		2021–2022			MVP candidates	MVP candidates
		2022–2023			All-stars	Stars
	P5	2017–2018	C	20	Stars	Stars

M, mean; SD, standard deviation; FSD, full-stack developer; GD, graphic designer; P, player; SF, small forward; G, Guard; PF, power forward; C, center.

### 2.3 The mobile application: System architecture and workflow

When a user initiates an action via the client-side interface—through the mobile application that targets the REST API layer—the inbound HTTP request is immediately intercepted by the backend framework's route layer.

**Figure 2:**
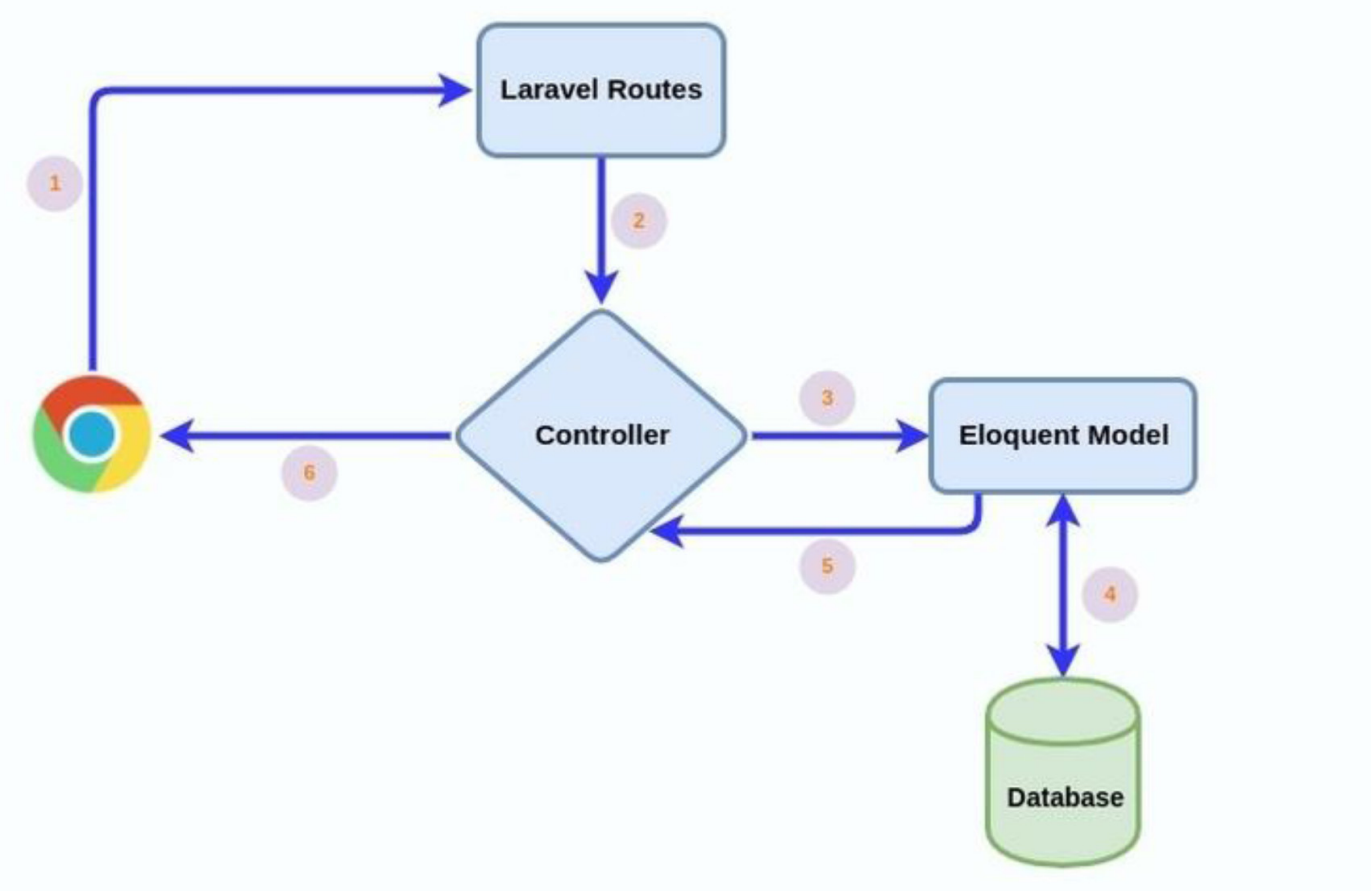
System architecture and request-response lifecycle.

As illustrated in Figure 2, the sequential lifecycle of a database request executes across six operational stages:

Stage 1: The user request is transmitted to the server architecture, where it is successfully captured and filtered by the dedicated route layer.

Stage 2: The route layer authenticates the inbound request and forwards it to the corresponding controller class responsible for handling the specific endpoint logic.

Stage 3: The controller processes the request payload, extracts key parameters, and invokes the appropriate model class to initiate the database query.

Stage 4: The model compiles and executes the SQL query within the database management system (DBMS), which then processes the query and returns the raw relational data to the model.

Stage 5: The model maps and hydrates these raw database records into structured data objects (ORM entities) and passes them back to the calling controller.

Stage 6: The Controller executes any final business logic or data transformation on the structured response before packaging it into a JSON payload and transmitting it back to the client application interface.

### 2.4 Data collection

#### 2.4.1 Data collection in the planning phase

The data collection for the planning phase adopted a user-centered approach, beginning with a comprehensive literature review, followed by an analysis of users' digital characteristics and basketball performance statistics. To establish efficiency norms, performance data for all young male players in the Turkish Basketball Youth League (BGL) were retrieved from the Turkish Basketball Federation's official website at the end of the 2021–2022 season. The consistency of norms was assessed by independent and volunteer coaches (C) and former players (FP) who were selected as experts. Additionally, the functional features of two existing basketball statistics platforms were analyzed. This phase concluded with structured interviews with the software developer and logo designer to align technical and visual requirements.

#### 2.4.2 Data collection in the developing phase

During the development phase, data collection focused on the application's technical architecture. The software developer detailed the technologies and frameworks used during regular alignment meetings. All technical specifications and design data were documented in text format for systematic review.

#### 2.4.3 Data collection in the improving phase

During the 2022–2023 season, qualitative usability data were collected using a mixed-methods nested sampling approach. Based on the prior quantitative norm analysis, five players were selected via maximum variation sampling to represent different norm groups.

The moderated usability tests were conducted online via Zoom, averaging 30 min per session, and yielded 30 pages of transcription. Prior to the sessions, informed consent was obtained from all participants (and their legal guardians, where applicable), ensuring anonymity through nicknames and confirming their right to withdraw at any time. To enhance reliability, sessions recorded both the participants' video/audio and their on-screen interactions, which were independently reviewed by a second researcher.

The usability test protocol was developed based on the user experience literature and expert feedback, incorporating both quantitative and qualitative questions, as well as specific task scenarios. In this process, the qualitative usability test was developed based on feedback from five experts. To familiarize players with the process, a pre-task (account creation) was introduced. The testing utilized a concurrent think-aloud technique (Çelik et al., 2023) to capture real-time cognitive processes and usability challenges. Participants were systematically prompted to verbalize their thoughts throughout the tasks, in strict accordance with established methodological recommendations for usability testing in educational and sports technologies.

Specifically, the testing protocol required the execution of four core, scenario-based tasks:

Task 1 (Onboarding/Installation): Download and install the application from the shared file or click the provided link to register using personal credentials, then log in to the interface.

Task 2 (Dashboard Navigation): Explore the primary dashboard and freely navigate through the interface to assess layout familiarity and visual ergonomics.

Task 3 (Social Comparison): Access the peer performance matrices to compare individual statistics against established team and league norms.

Task 4 (Goal Setting): Utilize the goal-setting interface to establish two distinct, individualized performance targets based on personal statistical metrics.

### 2.5 Data analysis

#### 2.5.1 Data analysis in the planning phase

The planning phase data analysis includes analysis of the literature, users' digital characteristics, norm development, competitor analysis, and stakeholder interviews.

Although the literature review continued throughout the research, the analysis of the theories and components to be used in the application's design was completed at the planning stage. And then, players' digital characteristics were compiled. The efficiency norms were developed using data from all young male players in the Basketball Youth League. Competitor (similar) apps that are commonly used in Türkiye by basketball players and coaches were examined in the competitor analysis.

Document analysis (Özkan, 2023) was used to explore the theories and components to be used in the application's design, as well as the digital characteristics of the mobile app's target audience. Team development linear theory, goal-setting theory, cognitive evaluation theory, intrinsic motivation, the Fogg behavior model, and social comparison theory were reviewed to explore the main theoretical frameworks underpinning the development of the mobile application. For the digital characteristics of the mobile app's target audience, the “Digital 2023: Turkey” report (Kemp, 2023) was analyzed.

Descriptive statistics (means (M), standard deviations (SD), minimum, and maximum scores) were used for players' characteristics, while players' efficiency scores were used as a performance parameter. Turkish and Euroleague basketball use the Performance Index Rating (PIR), the official box-score rating standard, as a player's efficiency score to measure a player's overall contribution throughout a game. PIR formula is below:

PIR = (Points + Rebounds + Assists + Steals + Blocks + Fouls Drawn)–(Missed Field Goals + Missed Free Throws + Turnovers + Shots Rejected + Fouls Committed)

Since players' match counts varied, efficiency scores were standardized by dividing each score by the number of matches.

The Rankit Z-score method was used to develop the efficiency scores' norms, following the steps recommended by (Erkuş and Selvi 2019).

1. Providing proofs of validity and reliability for the measurement tool.
2. Determination of a sample that reflects the infrastructure and influencing variables of the measurement tool and represents the universe.
3. Data collectors collect data within the framework of a certain standard and identify the variables that are likely to affect the data.
4. Careful processing of data, checking for missing values and outliers, and correcting if necessary.
5. Examining the raw score distributions separately according to the control variables, collecting data again if necessary.
6. Examining the control variables.
7. Finding the cumulative frequency of the raw scores for each level,
8. Regulating the distribution.
9. Converting the regulated data to percentile scores and/or standards, plotting the regulated score or the converted score graph to verify after conversion.
10. Comparative raw score-norm value tables are reported for each variable.

All data were checked for outliers using the Z-score method, a univariate outlier detection method (Akbaş and Kogar, 2020). Data outside the ±3 range were treated as outliers and excluded from the process. Then, both group-value-based and ranking-based analyses of norm development were conducted.

Before the norm development analysis, analysis of covariance (ANCOVA) and analysis of variance (ANOVA) were used to determine which variables (season and age) were significant for inclusion in the norm development process. Assumptions of normality, homogeneity of variance, linearity, reliable measurement of the covariate, and homogeneity of regression were checked for ANCOVA (Pallant, 2011). Furthermore, the assumption that the covariate and the independent variable share variance was assessed using ANOVA (Can, 2016). Non-normally distributed efficiency score data were transformed (using the square root technique) for parametric analysis (Pallant, 2011). The efficiency score variable was homogeneous for seasons and ages (*p* < 0.05; Levene's test of homogeneity of variances). There was no violation of the assumptions of linearity, homogeneity of regression slopes, and reliable measurement of the covariate for ANCOVA. Age (years) and playing time (seconds) were examined as covariates in the efficiency scores. However, age was not included as a covariate in the season comparison because it differed significantly between seasons. Playing time (sec) was used as a covariate for the season's comparison. In the age-group comparison, the playing time was checked as a covariate for efficiency scores. However, it was not used as a covariate because it shared variance with the age group variable. As a result, it was excluded from the analysis, and ANOVA was used for the comparison. Assumptions of normality and homogeneity of variance were checked for ANOVA (Pallant, 2011).

Non-normally distributed efficiency score data were transformed (using the square root technique) for covariance analysis by seasons (Pallant, 2011). As a result of this transformation, the number of samples decreased in this analysis because some negative efficiency scores were not assigned values, yielding a final sample of 1,477 players. The efficiency score variable was homogeneous for age comparison (*p* < 0.05; Levene's test of homogeneity of variances). Tamhane was used for multiple comparisons between age groups. Cohen's (1988) effect sizes (ESs) and thresholds (0.00, 0.06, and 0.14 as benchmarks for small, medium, and large effect sizes, respectively) were used to compare the magnitude of differences in efficiency scores across season, age, and position groups. The partial eta squared (Cohen, 1988) was used to determine the proportion of variation in players' efficiency scores explained. By multiplying the partial eta squared value by 100, it was converted to a percentage (Pallant, 2011). The Statistical Package for the Social Sciences (SPSS 26^®^) software was used for statistical analysis, with a significance level of 0.05 and a 95% confidence interval (CI).

The quantitative content analysis (Bilgin, 2014) was used to analyze the competitor apps (TBF website/app and the InStat Basketball Scout App).

#### 2.5.2 Data analysis in the developing phase

During the development phase, all information from the developer was transferred to the computer in written form. Then, the technologies used by the software developer were presented.

#### 2.5.3 Data analysis in the improving phase

In the data analysis for this phase, the developer's raw (unprocessed) data were transferred to the computer in written form. Then, these recordings were transcribed and analyzed with Microsoft Word. Content analysis was used for the qualitative analysis in this research (Bilgin, 2014). Content analysis is the process of grouping similar data within a dataset according to specific concepts and themes and presenting the results to readers in a clear and understandable manner. Content analysis consists of the following phases: coding data, organizing codes and themes, and defining and interpreting findings (Yildirim and Simşek, 2011). The researcher followed these phases during the qualitative data analysis. To ensure the trustworthiness and rigor of the coding procedure, an inductive line-by-line coding approach was utilized. Initial codes were generated independently by the primary researcher and an external qualitative analysis expert. Discrepancies between the coders were resolved through peer debriefing and consensus sessions until a 100% agreement on final themes was achieved.

Furthermore, the researcher observed the participants' preferences and behaviors by rewatching their Zoom recordings while using the application. These behavioral observations (e.g., navigation patterns) were systematically presented in the joint display table and triangulated with textual codes derived from the transcripts to form a comprehensive qualitative matrix. The qualitative usability test, combined with survey questions, enabled the simultaneous collection of both quantitative and qualitative data. Survey questions were comprised of 5 questions, each of which was rated on a 5-point scale ranging from 1 to 5 (1 = Very Poor; 2 = Below Average; 3 = Average; 4 = Above Average; 5 = Excellent). Therefore, descriptive statistics (means (M), standard deviations (SD), and minimum and maximum scores) were also used in this phase for the players' opinions on the app design.

### 2.6 Validity and reliability

#### 2.6.1 Validity and reliability in the quantitative analysis

The 10 stages recommended by (Erkuş and Selvi 2019) were used in the norm development process and served the construct validity for the developed player efficiency norms. In other words, executing this structured 10-stage progression ensures that the final transformed percentile distributions, standardized Z-scores, and assigned performance badges function as highly stable, consistent, and scientifically sound reference points for youth basketball evaluation.

Furthermore, 10 basketball coaches and 5 former players contributed domain-specific expertise on the norm-development process for performance evaluation and consistency (Table 2).

**Table 2:** Demographics of the coaches and former players for the norms' consistency process.

Codes	Gender	Education level	Basketball expertise	League	Season
C1	Male	Bachelor	E	BYL	2017–2018
C2	Male	Master of science	A	BYL	2017–2018
C3	Male	Master of science	B	U16 national coach	2020–2021
C4	Male	Bachelor	C	BYL	2019–2020
					2020–2021
C5	Male	Bachelor	D	BYL	2020–2021
C6	Male	Bachelor	C	BYL	2021–2022
C7	Male	Bachelor	C	BYL	2021–2022
C8	Male	Bachelor	A	BYL	2017–2018
					2018–2019
C9	Male	Bachelor	C	BYL	2017–2018
C10	Male	Bachelor	B	BYL	2018–2019
FP1	Male	Bachelor	PG	BYL	2017–2018
FP2	Male	Bachelor	SF/PF	BYL	2017–2018
					2018–2019
FP3	Male	Bachelor	PF/C	BYL	2019–2020
FP4	Male	Bachelor	PG/SG	BYL	2020–2021
FP5	Male	Bachelor	PF/C	BYL	2021–2022

C, Coach. Basketball coaching in Türkiye is divided into five categories: E, D, C, B, and A. FP, formal player; PG, point guard; SG, shooting guard; SF, small forward; PF, power forward; C, Center.

Some of them shared their opinions on norms and performance consistency. Some of these opinions are given below. Some of these opinions are given below:

C2: “*MC and AA may be in the MVP (Most Valuable Player) norm. They were invited to the youth national team try-outs that season. GG could be an All-Star. He played a very good season. CS was an Average Player. Others are consistent.”*

C6: “*Age is an important factor for BGL. In terms of seasons, the norms are consistent with the players*' *performances. However, statistics alone are not enough to assess players*' *defensive patterns. For example, OC, one of our team*'*s players, used to handle defensive duties. He usually had the role of defending the opponent*'*s top scorers and aimed to keep them below their standard performances. Although I admit that OC is not the norm for all-stars in his performance, I think his efficiency score should be 6 or 7 (he emphasizes that the efficiency score does not adequately evaluate defense). But overall, the players*' *performances and norms are consistent.”*

C7: “*Your values (norm scores) are a clear shot (consistent) when I think of long-term training and match performances.”*

C8: “*Star groups are very large (The range of the Stars is very wide), there are players who should not be there when compared to others.”*

FP1: “*I'd say inconsistent for one out of twelve in our team. Overall, I think my teammates' performances are consistent with the norm. When I think about my own performance, I didn't have enough playing time because I was a rookie. So, it's normal for my efficiency score to be low. In short, this norm group consists of my performance.”*

FP2: “*Overall, I think the norms are consistent with performances. Considering my own performance, I think it's normal for me to be in these norms for both seasons.”*

FP3: “*I think it does not reflect (it is not consistent), the scouting is necessary, for example, I was a man of duty (he means the need for observation of defense responsibilities). Furthermore, I think the roles that coaches give to players are important for this statistical evaluation. Therefore, I think this kind of statistical evaluation is insufficient.”*

FP4: “*Yes, it seems very realistic (He means that the norms and performances are consistent).”*

In one of the views, it was emphasized that the Stars norm has a wide range. The stars' norm is the group within ±1 according to their Z-scores. The fact that this range in norm development was noticed by the experts shows that the experts whose opinions were taken made a detailed evaluation. This was noted earlier in the research process, and percentiles were used for performance and to develop Z-score-based norms.

In another view, it was emphasized that age was important for BGL. The coach's opinion supported the significant difference found between age groups in the norm-developing analysis and the need to develop norms for each age group.

Coaches and players who gave their views stated that the norms and performance were generally consistent. On the other hand, the comments regarding the inconsistency between norms and performances draw attention to the fact that player statistics are insufficient for presenting defensive performance, rather than for assessing consistency between performances and norms. In other words, these opinions show that there were few opinions.

#### 2.6.2 Validity and reliability in the qualitative analysis

Credibility (in place of internal validity), transferability (in place of external validity), dependability (in place of reliability), and confirmability (in place of objectivity) are used for the validity and reliability processes of the qualitative analysis (Lincoln Yvonna and Guba Egon, 1985). The researcher played a proactive role throughout the research and employed multiple strategies (data, method, and researcher triangulation, expert opinion, and rich descriptions) to guide these processes. Furthermore, the researcher attended courses taught by both quantitative and qualitative research experts and incorporated their feedback into the research process.

The qualitative usability test was developed with the opinions of experts (field experts, developers, and researchers). In the qualitative usability test development process, the planning phase of the design, user experience research literature, and think-aloud technique literature were also utilized. This test was developed as a moderated test (both remote and face-to-face) and included various question types (quantitative and qualitative) and many tasks. Furthermore, a pre-task (create-account task) was added to familiarize participants with the test and the think-aloud technique. The players were reminded to use the think-aloud technique between tasks. In addition, the data were transcribed immediately after the interviews. In other words, recommendations for using the think-aloud technique in a qualitative usability test (Çelik et al., 2023) were followed during data collection.

In addition, recording videos of the participants and their screens as they used the application allowed the researcher to observe their behaviors and choices again. Therefore, the test allowed the researcher to obtain more reliable data and to conduct data triangulation. These steps increased credibility (in place of internal validity), transferability (in place of external validity), and dependability (in place of reliability). During data collection, participants' verbal consent was obtained for the interview transcripts. A volunteer researcher was asked to check both the audio and video records of the interviews to confirm the accuracy of the transcripts. During the coding phase, the researcher compared the qualitative results and determined the codes they agreed or disagreed with.

All statistics of the target population were used for norm development, while maximum variation sampling was used for the interviews. Given the quantitative and qualitative sampling methods used in the research, nested sampling was employed as a mixed-methods sampling approach. These sampling methods increased transferability (at the expense of external validity).

In the research, the characteristics of the participants and experts, the development process of the data collection tools, and the data analysis were presented in rich detail, thereby increasing the research's confirmability.

#### 2.6.3 Validity and reliability in the mixed methods integrative analysis

In this design-based research method, which adopts a mixed-methods approach, paradigmatic mixing and commensurability (Onwuegbuzie and Johnson, 2006) were used to support validity and quality processes. While paradigmatic mixing is the successful combination or blending of quantitative and qualitative methods, along with epistemological, ontological, axiological, methodological, and rhetorical beliefs, into a usable package, commensurability is the ability to make meta-inferences by constantly switching between quantitative and qualitative methods (Onwuegbuzie and Johnson, 2006).

As a paradigmatic mixing strategy, meta-inference was used to integrate quantitative and qualitative analyses conducted during the planning, development, and improvement phases of the design-based research methodology. During the use of the constant comparison technique, memoing assisted this process by prompting reflection on shifts in paradigmatic assumptions as meta-inferences were created.

In terms of commensurability, this research utilized fully integrated analyses that link, combine, and interrelate the planning, development, and improvement phases. In terms of commensurability, this research utilized fully integrated analyses that link, combine, and interrelate the planning, development, and improvement phases.

The quantitative data, supported by qualitative expert opinions and the qualitative usability test, combined with survey questions, enabled the collection of different data types at different stages of the research.

In addition, the researcher, who received support from many stakeholders (players, norms consistency experts, logo designer, developers, etc.), used both quantitative (norm development) and qualitative (user experience tests, etc.) methods and data. In other words, the researcher employed theory triangulation, method triangulation, and data triangulation (Denzin, 2017), drawing on various theories, methods, investigators, and data throughout this research.

## 3 Results

### 3.1 Planning phase

In this phase, the literature was reviewed, the players' digital characteristics were compiled, efficiency norms were developed, and competitors were analyzed. At the end of this phase, all results were shared with stakeholders.

#### 3.1.1 Literature review for the application design

**Figure 3:**
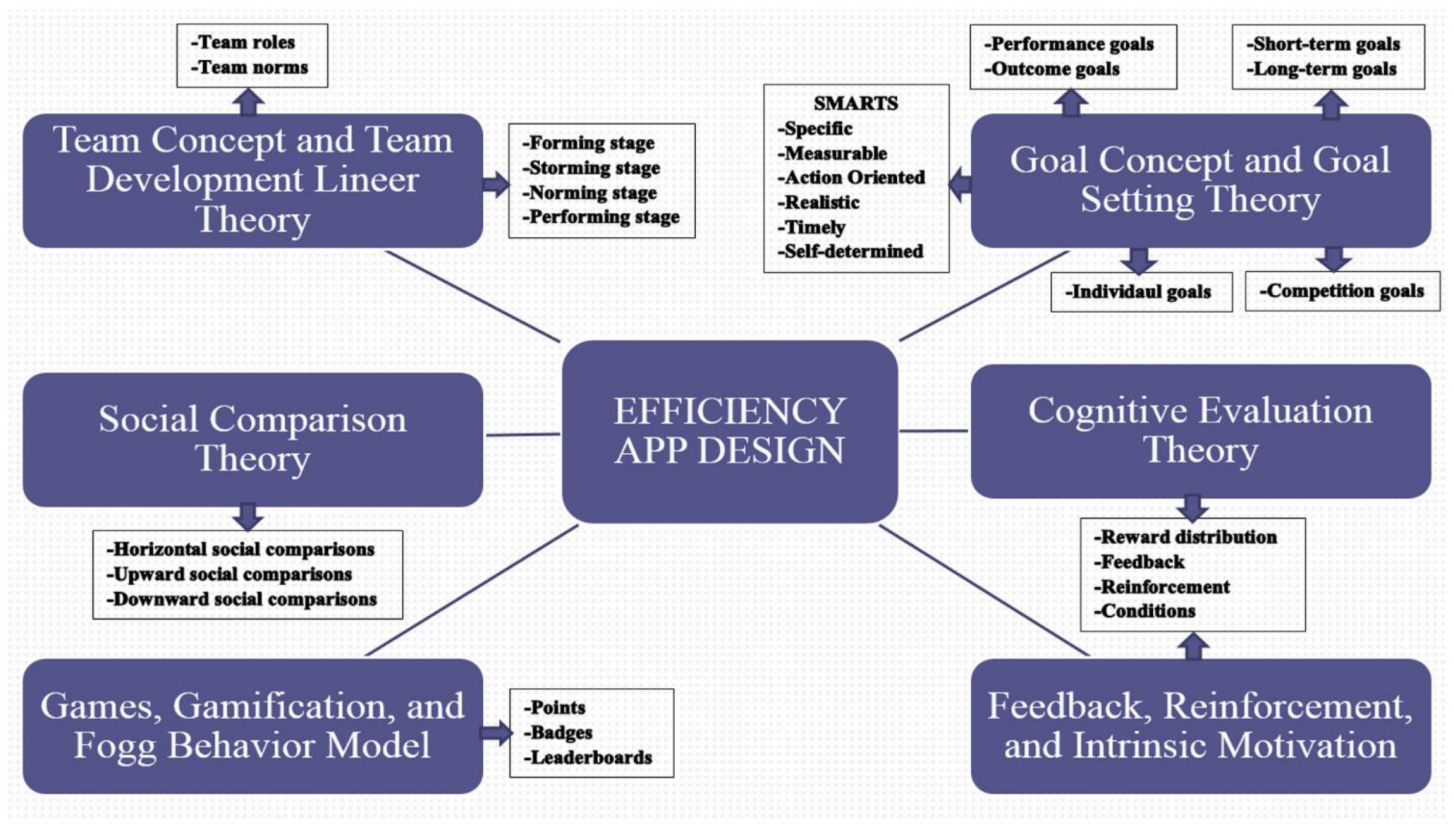
Mobile app theoretical design.

According to the literature review, the main theoretical frameworks used in the development of the mobile application include team development linear theory, goal-setting theory, cognitive evaluation theory, intrinsic motivation, the Fogg behavior model, and social comparison theory (Figure 3).

#### 3.1.2 Digital characteristics of the target users

According to the document analysis of players' digital characteristics, individuals aged 16 to 64 increasingly prefer mobile technologies for connecting to the Internet each year. The reasons for using the Internet and social media in the report were examined, and some of the main reasons for using the Internet by individuals aged 16–64 were finding information (73.2%), keeping up to date with news and events (67.6%), staying in touch with friends and family (58.3%), and education and study-related purposes (49.5%).

#### 3.1.3 Norm development for players' efficiency ratings

The development of player efficiency norms was guided by the comprehensive 10-stage methodological framework. Within this systematic norm development process, descriptive and variance analyses played pivotal roles during the initial stages. Descriptive analysis was used to examine the raw score distributions separately by the control variables to establish the operational parameters for stratification. Before generating the final norm matrices, analysis of variance (ANOVA) and analysis of covariance (ANCOVA) were executed to empirically determine which demographic and contextual variables (such as age groups and competitive seasons) significantly influenced player efficiency scores. These preliminary statistical steps provided the necessary empirical justification for the framework's transition from a universal target population to stratified subpopulations. Consequently, the variance analysis findings necessitated the creation of age- and season-specific stratified norm matrices, ensuring that subsequent stages of standardized score conversion (Z-scores and percentiles) and the final assignment of performance profile badges were based on a scientifically validated structural framework.

Raw score distributions were described separately by the levels of the control variables (Table 3). Due to the limited sample size in certain age subgroups, 15- and 16-year-old players were combined with 17-year-old players, and 20-year-old players were combined with 19-year-old players. Then, analyses were performed for control variables. After the recoding, the number of 15-, 16-, and 17-year-old players was 651. The number of 18-year-old players was 600. The number of 19- and 20-year-old players was 305.

**Table 3:** Descriptive statistics of the players' efficiency according to the season and age.

Control Variables	Subgroups	*n*	M	SD	Min	Max
Seasons	2017–2018	300	6.14	5.21	−1.60	27.00
	2018–2019	303	5.69	5.10	−2.00	29.00
	2019–2020	311	6.43	5.93	−2.69	47.00
	2020–2021	304	5.80	5.50	−5.00	29.25
	2021–2022	338	5.79	5.28	−2.00	27.67
Ages	15	2	1.75	1.77	0.50	3.00
	16	98	3.63	4.53	−2.00	29.00
	17	552	3.80	3.95	−5.00	26.66
	18	599	5.85	4.90	−3.00	47.00
	19	277	10.70	5.57	−4.00	23.80
	20	28	12.96	6.80	1.86	29.25

M, mean; SD, standard deviation.

Following the descriptive analysis, seasons and age were examined to determine whether they were significant variables in norm development. According to the covariance analysis results, controlling for season, there was a significant relationship between players' efficiency score and minutes played. Meaning players' efficiency ratings are affected by the minutes they play (*F*_(1, 1471)_ = 3,332.852; *p* < 0.000; η^2^ = 0.694). After adjusting for minutes played of players, there was no significant difference between seasons (*F*_(4, 1471)_ = 1.807; *p* = 0.125; $\eta_{\text{p}}^{2}$ = 0.005; Table 4).

**Table 4:** Covariance analysis for efficiency score by seasons.

Source	SS	*df*	MS	*F*	*P*	$\eta_{\text{p}}^{2}$
Minutes played (Sec)	1,186.862	1	1,186.862	3,332.852	< 0.001^***^	0.694
Seasons	2.575	4	0.644	1.807	0.125	0.005
Error	523.838	1,471	0.356			
Total	1,717.946	1,476				

^***^p < 0.001; *p* > 0.05; SS, sum of squares; *df* , degrees of freedom; MS, mean square; $\eta_{\text{p}}^{2}$, partial eta squared.

Conversely, according to the ANOVA results, there was a significant difference in player ages (*F*_(2, 1553)_ = 235.834; *p* < 0.000; η^2^ = 0.233). Tamhane's multiple comparison tests showed that 19/20-year-old players had higher performance than 18-year-old players (*p* < 0.001) and 15/16/17-year-old players (*p* < 0.001). It also showed that 18-year-old players had higher performance than the 15/16/17-year-old players (*p* < 0.001; Table 5).

**Table 5:** One-way analysis of variance for efficiency scores of players by age groups.

Age groups	*n*	M ±SD	95% CI	*F*	*p*	$\eta_{\text{p}}^{2}$	Tamhane
15/16/17	651	3.77 ± 4.04	3.46–4.08	235.834	< 0.001^***^	0.233	19/20 > 18^***^
18	600	5.85 ± 4.90	5.45–6.24				19/20 > 15/16/17^***^
19/20	305	10.91 ± 5.72	10.27–11.56				18 > 15/16/17^***^

^***^p < 0.001.

M, mean; SD, standard deviation; CI = 95% Confidence Interval for the mean (Lower Bound - Upper Bound); $\eta_{\text{p}}^{2}$ = partial eta squared.

While a single norm and percentage were reported for the seasons, as they did not differ, three age norms and percentages were reported because the age groups differed. The norms were named and used as badges, inspired by the players' achievements. Percentile intervals (1–99%) illustrate the cumulative distribution across different age groups (15/16/17-year-olds, 18-year-olds, 19/20-year-olds) and overall seasons (Table 6).

**Table 6:** Norms and percentages of efficiency scores according to the seasons and ages.

Parameter	Z scores	Efficiency score range (*n*)	Badges	Percentage (%)	Efficiency score limits (*n*)	Badges
Seasons	(−3)–(−2)	(−3.00)–(−0.75) (33)	Rotation Players	1–10	(−3.00)−0.5 (175)	
				11–20	0.53–1.42 (148)	
	(−2)–(−1)	(−0.67)–(1.00) (223)	Average Players	21–30	1.43–2.33 (156)	
				31–40	2.35–3.33 (153)	
	(−1)–(+1)	1.04–11.24 (1,044)	Stars	41–50	3.35–4.67 (155)	
				51–60	4.68–6.26 (153)	National player candidates
	(+1)–(+2)	11.25–18.36 (210)	All-stars	61–70	6.26–8.17 (155)	
				71–80	8.18–10.32 (154)	
	(+2)–(+3)	18.43–21.38 (33)	MVP candidates	81–90	10.34–13.79 (155)	
				91–99	13.84–21.38 (137)	
19/20-year-old players	(−3)–(−2)	(−4)−0.33 (7)	Rotation players	1–10	(−4)−3.64 (33)	
				11–20	3.67–6.38 (31)	
	(−2)–(−1)	0.63–5.14 (41)	Average players	21–30	6.58–7.80 (30)	
				31–40	7.88–9.13 (31)	
	(−1)–(+1)	5.23–16.75 (208)	Stars	41–50	9.24–10.69 (30)	
				51–60	10.08–12.47 (30)	
	(+1)–(+2)	16.8–21.07 (41)	All-stars	61–70	12.47–14.06(31)	
				71–80	14.11–16.39 (30)	
	(+2)–(+3)	21.38–27.66 (7)	MVP candidates	81–90	16.40–18.96 (31)	
				91–99	19.09–27.66 (27)	
18-year-old players	(−3)–(−2)	(−2.69)–(−0.75) (12)	Rotation players	1–10	(−2.69)–(0.65) (64)	
				11–20	0.67–1.69 (60)	
	(−2)–(−1)	(−0.50)−1.27 (82)	Average players	21–30	1.70–2.78 (61)	
				31–40	2.80–3.75 (60)	
	(−1)–(+1)	1.33–10.58 (408)	Stars	41–50	3.8–4.96 (59)	
				51–60	5.00–6.41 (60)	
	(+1)–(+2)	10.61–16.31 (81)	All-stars	61–70	6.42–8.08 (60)	
				71–80	8.15–9.67 (59)	
	(+2)–(+3)	16.56–20.03(13)	MVP candidates	81–90	9.69–12.56 (60)	
				91–99	12.59–20.03 (53)	
15/16/17-year-old players	(−3)–(−2)	(−2.00)–(−1.00) (17)	Rotation players	1–10	(−2.00)−0.00 (75)
				11–20	0.09–0.82 (60)
	(−2)–(−1)	(−0.75)−0.50 (91)	Average players	21–30	0.83–1.39 (64)
				31–40	1.40–2.1 (66)
	(−1)–(+1)	0.53–7.06 (436)	Stars	41–50	2.11–2.74 (63)
				51–60	2.75–3.50 (66)
	(+1)–(+2)	7.07–13.56 (88)	All-stars	61–70	3.54–4.59 (65)
				71–80	4.62–6.38 (65)
	(+2)–(+3)	13.67–21.08 (14)	MVP candidates	81–90	6.42–9.00 (65)
				91–99	9.06–21.08 (57)

Efficiency score ranges are categorized based on standardized Z-scores, which correspond to distinct player performance profile badges: Rotation Players (−3 to −2), Average Players (−2 to −1), Stars (−1 to +1), All-Stars (+1 to +2), and MVP Candidates (+2 to +3). Moreover, one badge is categorized by percentile intervals and is named the National Player Candidates badge, as it represents the percentile interval within which players are selected for national teams.

#### 3.1.4 Competitor analysis

Responsive design, feedback (notifications), and social comparison features are present (+) in all three applications (Table 7). Web 2.0 functionality is integrated into both the Instat Basketball Scout App and Stats & Goals, but is absent (–) in the TBF App. Conversely, video analysis is exclusively available in the Instat Basketball Scout App and is not featured in either the TBF app or Stats & Goals. Gamification elements are present (+) in the TBF App and Stats & Goals, while being absent (–) from the Instat Basketball Scout app. Finally, the goal-setting feature is uniquely present (+) in Stats & Goals, whereas it is absent (–) in both the TBF app and the Instat Basketball Scout app.

**Table 7:** Competitor analysis.

Features/Apps	TBF app	Instat basketball scout app	Stats & goals
WEB 2.0	−	+	+
Responsive design	+	+	+
Feedback (notifications)	+	+	+
Goal setting	−	−	+
Social comparison	+	+	+
Gamification	+	−	+
Video analysis	−	+	−

#### 3.1.5 Interviews with the stakeholders

**Figure 4:**
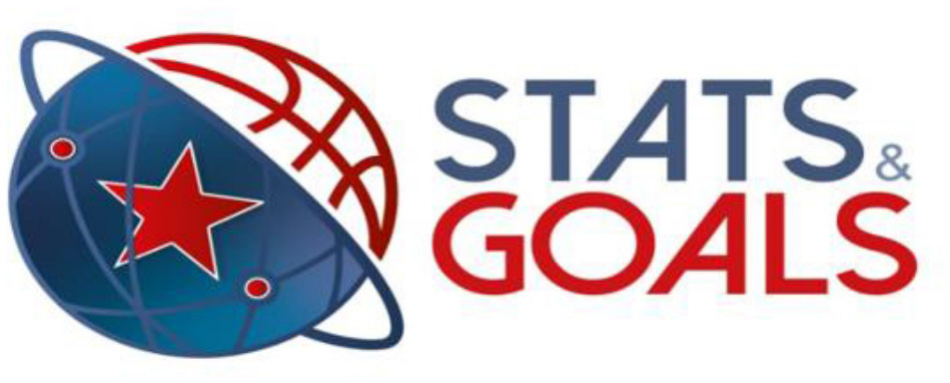
Logo of stats & goals.

After the interviews with GD, GD designed 5 logo samples. One of them was selected as the app's logo by the researcher, other researchers, and FSD (Figure 4).

### 3.2 Developing phase

The technical architecture, programming languages, and development frameworks used during the iterative development phase of the mobile application are systematically organized into specific operational categories (Table 8).

**Table 8:** Technical infrastructure and frameworks of the development phase.

Operational role & workflow functionality	Technical category	Technology/component
1. Design phase: wireframe and layout prototyping to define user interface requirements.	Interface design	UI/UX Mockups
2. Data layer: relational database management system for software infrastructure and data storage.	Database technology	MySQL
3. Backend Language: Server-side language used to build database communication and business logic.	Programming language	PHP
4. Backend Framework: Framework utilized for backend core, API routing, and database synchronization.	Backend framework	Laravel
5. Communication Layer: Architectural style for database-to-application communication and frontend execution.	System architecture	REST API
6. Frontend Language: Core programming language for client-side application development.	Programming language	Dart
7. Frontend Framework: A hybrid mobile application development framework for cross-platform rendering.	Development framework	Flutter

### 3.3 Improving phase

#### 3.3.1 Qualitative results

After creating the first app design, a qualitative usability test with different tasks, semi-structured interview questions, and survey questions was conducted to explore players' opinions and experiences of the mobile app. Qualitative data were presented by theme using an inductive, holistic perspective (Table 9).

**Table 9:** Themes of app opinions and experiences of the players.

Themes	Categories	Codes
WEB 2.0 app	Individualized app	– Log in – Sign up
User-friendly app	Ease of use	– Easy login – Simple design – No need to match with my information
Personalized app	Personal information	– Teams – Players – All seasons – My profile
	Performance statistics	– Match statistics – Tracking own statistics – Confusing statistics – Detailed statistics – Objective statistics
Customized app	Social comparison	– Comparing your own performance with yourself – Comparing your own performance with teammates – Comparing your own performance with similar players – Comparing your own performance with rivals
	Goal setting	– Goal setting – Optional goal types – Optional setting different goals – Self-determined goals – Saving goals
Notification	Feedback	– Feedback – Achieving a goal or not
Indirect advantages	Team building	– Positive effects on the team
	Guiding basketball stakeholders	– Guide basketball coaches – Guide managers
	Selecting players	– Selecting and transferring players
Suggestions	Player search tab	– Tab for searching players
	Easy login	– Player position categorization
	Familiar interface design	– Similar design to the match score sheet
	Personal photos and formal logos	– Player photos and team logos to recognize them
	Visible and editable goals	– Visible goal descriptions and editable goals
	Training programs for goals	– Roadmaps for achieving goals

Players were asked to sign up for the app in the first task of the qualitative usability test. The fact that players create accounts to use the application indicates that it is a WEB 2.0 application. In line with this, one of the players, P2, voiced that “*... I create an account by filling in my name and surname. I filled in my position. I filled in my weight. I filled in my height and signed up. I logged in.”* In this context, while there was the “individualized app” category under the “WEB 2.0 app” theme, there were “log in” and “sign up” codes under this category.

Furthermore, players reported easy access to the application during the first task and the ability to view their statistics directly in their accounts. In line with this, one of the players, P1, voiced that “*...I was able to log in easily. Nothing else caught my eye. So, I could log in directly.”* And P2 said, “So it's an easy login. The home page is already in front of us. When we entered our email and password, we were able to log in easily.” In this context, while there was the “ease of use” category under the “user-friendly app” theme, there were “easy login,” “simple design,” and “no need to match with my information” codes under this category.

Players were asked to surf the app during the second task of the qualitative usability test. Players said they had direct access to their personal information and performance statistics in the app. In line with this, one of the players, P1, stated that “…* When I click on the players, they are presented directly from here as a ranking. I think I'm in my profile right now. All seasons. There is also the seasons' section. Here I can choose a season and find out the season I played in. Nice features.”* and “*I think the player can check their own statistics. In other words, he can compare himself from one season to another season, or if he has another rival, he can compare himself with him who plays in the same position. Or he can use it to improve himself. The player can see his pros and cons.”* and “*… My profile appears directly when I log in, without selecting it. I don't have to do anything to choose myself in the profile section anyway.”*

The other player, P2, commented that “*…what my eye really caught were the teams right now. I'm looking at the BGL teams of the 2022–2023 season. They caught my attention. And the part right above it that was written “all seasons” button caught my attention. I can go back to the season I played. I can see the teams in the season I played; these are good features. The things I liked. As far as I can see, there are player statistics, I mean, player profiles at the bottom. It is formed in alphabetical order. These are also good features of the app.”* In this context, there were the “personal information” and “performance statistics” categories under the “personalized app” theme. While there were “teams,” “players,” “all seasons” and “my profile” codes under the “personal information” category; there were “match statistics,” “tracking own statistics,” “confusing statistics,” “detailed statistics,” and “objective statistics” codes under the “personal statistics” category.

While players were asked to compare their performance with their peers in the third task of the qualitative usability test, they were given two goals to set in the fourth task. While the players emphasized that they could compare their performance with themselves, their teammates, players with similar characteristics, and their opponents during the third task, they stated that during the fourth task, they could set many types of goals according to their preferences and record and track these goals.

P1 commented that “*...compare with me. In other words, after choosing the player directly, there was a “comparison with me” button. I click it now. There is what we both did in the same season. It says how many minutes we played, how many rebounds we took, and how many scores we made in the game. All of them are written here, sir. I can directly compare myself with using all of them.”*

P2 stated that “*…I think he was an important player for our team that year. I chose a rival of mine. I compared myself with him. Here, the statistics for both players are presented. Minutes played and scores—these are important statistics for us.”*

P3 voiced that “*…let me tell you this way, I think it's a good thing that when we click the button, it immediately shows statistics. I even click it again, and it is presented directly. I think it's a nice thing (performance). As I said, even a person who does not know basketball can understand this quite clearly.”*

P1 stated that “*...When I clicked from the top of the left, my goals section appeared. Season goal. Here I can create a new goal for myself. I can write the goal type myself. It shows a season goal, a match goal, and a performance goal.”*

P2 voiced that “*I click my goals section. As the goal type, I chose seasonal. I chose the 3-point goal type. So, my goal here is actually to try more 3-point shots. I think my 3-point percentage should increase. I can write my goal. Yes, I wish my 3-point try were more. This is my first goal...”*

and “*…And I saved this goal as well. My 2 goals are now appearing in front of me on my goals page….”*

P3 stated that “*… I click the goal, and I write. I am choosing the goal types: match goal and season goal. I always like to go match by match. I don't think of it as a season. I am such a player...”*

In this context, the “social comparison” and “goal setting” categories fell under the “customized app” theme. While there were “comparing your own performance with yourself,” “comparing your own performance with teammates,” “comparing your own performance with similar players,” and “comparing your own performance with rivals” codes under the “social comparison” category; there were “goal-setting,” “optional goal types,” “optional setting different goals,” “self-determined goals,” and “saving goals.”

The themes of notification, indirect advantages, and suggestions were yielded with probe questions and semi-structured interview questions during the qualitative usability test. P1 voiced on the “notification” theme that “*There is also a section saying whether you achieved a goal or not. It is designed as a status. After the match, if I reached the goal that I wanted to achieve, I would write that it was achieved there. From here I can see what I achieved and what I didn't achieve...”*

In this context, there was the “feedback” category under the “notification” theme. There were “feedback” and “achieving a goal or not” codes under the “feedback” category.

Some of the players' comments were on the “indirect advantages” theme. P1 stated that “*...I think it's good for both the coach and the player. The players can use the app to improve themselves. In addition, coaches can also learn the characteristics of all the players on the opposing team.”*

P2 voiced that “*... such applications are very valuable for us, basketball players and basketball coaches.”* and “*... it will have a huge impact on every player. It affects both the player and the team. If the player develops himself, his team will progress.”*

P3 stated that “*…when teams and general managers are looking for players, they can also look at us from this application….”* and “*… thanks to such applications, some things are heard, seen, and known. Such apps are given more consideration during the transfer season.”*

In this context, the “team building,” “guiding basketball stakeholders,” and “selecting players” categories fell under the “indirect advantages” theme.

Some of the players' comments were on the “suggestions” theme. P1 interview note: “*It would be nice if there was a shortcut to search for players.”* Moreover, P2 stated that “*…after setting my goals, I would like to have a roadmap (training program) on how I can reach these goals.”* And P3 voiced that “*In my opinion, when we click directly on the positions while we sign up, for example, all the positions from one to five, etc., should be presented as a category. I think it would be better if it were presented this way. You know, it will be easier for the user, and it will look nicer.”* and “*… It would be easier if the statistics were presented as on the scoresheet of the match, but this is also nice….”* and “*…and let me say this, I think it would be nicer if the logo of the team, rather than a random photo, were used while surfing through the teams….”* and “*...and a goal-setting page...it would be better to see the descriptions of my goal there after I save my goal...”* and “*…I think it would be better if we put player photos. Because, you know, we know most of the people in basketball face-to-face. You know, we don't know their names...”*

In this context, there were the “player search tab,” “familiar interface design,” “personal photos and formal logos,” “visible and editable goals,” and “training programs for goal” categories under the “suggestions” theme. In summary, seven themes were yielded from the qualitative data. These were the Web 2.0 app, user-friendly app, personalized app, customized app, notification, indirect advantages, and suggestions.

#### 3.3.2 Quantitative results

Because the qualitative usability test was combined with survey questions, it provided both quantitative and qualitative data. Descriptive statistics of the players' opinions were presented by task (Table 10). The average and median for each task is above 4 (Task 1 = 4.60 ± 0.55–5; Task 2 = 4.00 ± 0.71–4; Task 3 = 4.40 ± 0.55–4; Task 4 = 4.00 ± 0.71–4). The overall design mean and median score for the application in the survey is 4.60 ± 0.55–5.

**Table 10:** Descriptive statistics of the players for survey questions in the qualitative user test.

Tasks	*n*	M	SD	MD	Min	Max
Task 1	5	4.60	0.55	5	1	5
Task 2	5	4.00	0.71	4	1	5
Task 3	5	4.40	0.55	4	1	5
Task 4	5	4.00	0.71	4	1	5
Overall design	5	4.60	0.55	5	1	5

M, mean; SD, standard deviation; MD, median.

#### 3.3.3 Mixed methods integrative results

To develop the application in accordance with the integrative nature of mixed-methods research, a joint display table was presented, emerging from the integrated analysis of quantitative and qualitative findings for the app development process. A joint display table of mixed-methods integrative results showed how quantitative and qualitative findings are combined in the mobile application development process. The entire process was expressed through the narrative technique above the Table 11.

**Table 11:** Joint displays for the application development process.

Design-based research (Powered by literature analysis, norm development, competitor analysis, user experiences (UX) research, think-aloud technique, content analysis, and mixed methods integrative analysis (joint displays and narratives by constant comparison technique)					
Planning phase			Development phase	Improvement phase	Mixed methods integration
– Literature analysis •Team development linear theory •Goal-setting theory •Cognitive evaluation theory •Social comparison theory •Intrinsic motivation •Fogg behavior model and gamification •Basketball statistics and player efficiency ratio			– Theory-based design – Badges for gamification – Designing drafts for the app interface – Database technologies – The operation of the application infrastructure	**Qualitative usability test** – Seven themes from qualitative data •WEB 2.0 app •User-friendly app •Personalized app •Customized app •Notification •Indirect advantages •Suggestions – Survey scores •Task 1 = 4.60 ± 0.55 •Task 2 = 4.00 ± 0.71 •Task 3 = 4.40 ± 0.55 •Task 4 = 4.00 ± 0.71 •Overall Design= 4.60 ± 0.55	– Stats & Goals was designed as a WEB 2.0 application in the development phase as a result of digital analysis of target users and competitor analysis findings in the planning phase. The fact that players create accounts to use the application indicates that it is a WEB 2.0 application. – During the planning phase, it was decided to use a design-based research methodology and adopt a user-centered design while designing the application. The improvement phase findings showed that players can access the app easily and view their personal data and performance statistics. These findings showed that Stats & Goals is a user-friendly, personalized app with a user-centered design, as planned. – Competitor analysis and literature analysis during the planning phase of the application guided the comparison of the performances of the players with themselves and their peers and set goals for their individual performances. Furthermore, the norms developed during the planning phase of the application were added to the “set a goal” feature, considering that they would support the players' goal setting. In line with this, the “compare with me” and “set goals” pages were created during the development phase. While the improvement phase findings show that the players use the “compare with me” feature comfortably, they stated that there is a need for improvements in the “set a goal” feature. – The norms which were developed during the planning phase of the application were used as badges and leaderboards in the context of gamification during the development of the application. The improvement phase findings showed that norms should be emphasized more in design. Therefore, info boxes were added to the acronyms for all stats and norms.
– Digital characteristics of the target users •WEB 2.0 •Android and iOS operating systems			– App development framework & Programming languages – User-centered hybrid app		
– No significant difference between seasons (*F*_(4, 1471)_ = 1.807; *p* = 0.125; η^2^ = 0.005) – Significant difference between the ages of the players (*F*_(2, 1553_) = 235.834; *p* = 0.000; η^2^ = 0.221)			– Gamification •Badges •Leaderboards		
19/20 > 18^***^ Tamhane 19/20 > 15/16/17^***^ 18>15/16/17^***^					
– Norms were developed for general and age variables (15/16/17–18-19/20)					
**5 norms**	**10 percentages**						
Rotation Players Average Players Stars All-Stars MVP Candidates	1–10 11–20 21–30 31–40 41–50 51–60 61–70 71–80 81–90 91–99	National Player Candidates			
– Norms and percentages have consistency					
**Competitors analysis** – WEB 1.0 & WEB 2.0 – Responsive Design – No feedback (notifications) – No goal setting – Social comparison – Unregular Gamification			– WEB 2.0 – Responsive Design – Optional feedback (notifications) – Goal setting – Social comparison – Gamification		
**Interviews with the stakeholders** – Name and domain hosting – Logo design			Stats & Goals		

^***^p < 0.001.

The mixed-methods integrative results indicate that the youth players exhibit five norms, as reflected in the efficiency scores across seasons and age groups. In the preliminary analyses to identify significant factors for the norms, there was no significant difference between the seasons, but there was a significant difference between the age groups. The experts' opinions showed that norms are consistent. One of the experts emphasized that age is an important factor for BYL during the planning phase of norm development. In other words, quantitative data are compatible with qualitative data. Therefore, age norms were developed based on group values. In other words, the app allowed players to determine both their performance in the overall classification and their place within their own age groups. In the measurement and evaluation literature, the app provided the opportunity to report both general and age-group-specific summative evaluations.

The mixed-methods integrative results showed that the application is useful and satisfying. Qualitative usability tests, combined with survey questions, were conducted during the development phase. Themes of the WEB 2.0 app, user-friendly app, personalized app, customized app, notification, indirect advantages, and suggestions were derived from the qualitative data through content analysis. The overall design score of the application was 4.60 ± 0.55, as determined from quantitative data using descriptive analysis. While qualitative data from the usability test showed that players found the application useful, quantitative data indicated that they were satisfied with it.

Stats & Goals was designed as a WEB 2.0 application during the development phase, based on digital analysis of target users and findings from the planning phase's competitor analysis. The fact that players create accounts to use the application indicates that it is a WEB 2.0 application. In line with this, one of the players, P2, proved that the application is a WEB 2.0 application by creating an account to use the application and expressed the process of creating an account to use the application: “*... I create an account by filling in my name and surname. I filled in my position. I filled in my weight. I filled in my height and signed up. I logged in.”*

During the planning phase, it was decided to use a design-based research methodology and adopt a user-centered design while designing the application. In this context, a user-centered design-based research method was employed during the application's development. The improvement phase findings showed that players can access the app easily and view their personal information and performance statistics. In other words, Stats & Goals is a user-friendly, personalized app. These findings show that the application is user-friendly and has a user-centered design as planned. In line with this, one of the players, P1, explained that the application is user-friendly, with the phrase: “*...I was able to log in easily. Nothing else caught my eye. So, I could log in directly.”* Another player, P3, explained that it was a personalized app with the phrase: “*… My own profile appears directly without selecting it when I log in to the profile. I don't have to do anything to choose me in the profile section anyway.”*

Competitor and literature analyses during the application's planning phase guided comparisons of players' performance against themselves and their peers, and the setting of goals for their individual performance. Furthermore, the norms developed during the planning phase of the application were added to the “set a goal” feature, considering that they would support the players' goal setting. In this context, “compare with me” and “set a goal” pages were created during the development phase. However, players preferred to use other stats rather than norms and tended to rely on previously familiar statistics rather than new ones. In other words, while the improvement phase findings show that players use the “compare with me” feature comfortably, they also indicate a need to improve the “set a goal” feature. In line with this, one of the players, P1, explained that for the “compare with me” feature, with this phrase: “*...compare with me. In other words, after choosing the player directly, there was a” compare with me “button. I click it now. There is what we both did in the same season. It says how many minutes we played, how many rebounds we took, and how many scores we made in the game. All of them are written here, sir. I can directly compare myself with using all of them.”*

Another player, P2, stated that for the “compare with me” feature: “*…I think he was an important player for our team that year. I chose a rival of mine. I compared myself with him. Here, the statistics for both players are presented. Minutes played and scores are important statistics for us.”*

P1 stated that for the “set a goal” feature: “*...When I clicked from the top of the left, my goals section appeared. Season goal. Here I can create a new goal for myself. I can write the goal type myself. It shows a season goal, a match goal, and a performance goal.”*

P3 offered that for the “set a goal” feature: “*...and goal-setting page...it would be better to see the descriptions of my goal there after I save my goal...”*

The norms developed during the application's planning phase were used as badges and leaderboards in the application's gamification. The findings from the improvement phase showed that norms should be emphasized more in design. Therefore, info boxes were added to the acronyms for all stats and norms. In line with this, one of the players, P3, explained with this phrase: “*…let me tell you this way, I think it's a good thing that when we click the button, it immediately shows statistics. I even click it again, and it is presented directly. I think it's a nice thing (performance). As I said, even a person who does not know basketball can understand this quite clearly.”*

## 4 Discussion

### 4.1 Planning phase

The initial phase of this study exemplifies the context-focused, theory-based, and pragmatic nature of Design-Based Research (DBR; Wang and Hannafin, 2005). To construct a solid conceptual framework for the mobile application, multiple theoretical frameworks were integrated, including linear team development, goal setting, cognitive evaluation, social comparison, the Fogg behavior model, gamification, and Web 2.0 architectures.

The application explicitly employs efficiency-based productivity norms (Munroe et al., 1999) to foster team cohesion in line with the linear team development (Weinberg and Gould, 2010).

In team sports, clarifying and accepting distinct structural responsibilities is paramount to enhancing group effectiveness, boosting satisfaction, and reducing social loafing (Beauchamp et al., 2005). By tracking and reinforcing these parameters digitally, the mobile application is designed to systematically guide youth squads through the sequential forming, storming, norming, and performing stages of development (Weinberg and Gould, 2010).

Furthermore, integrating social comparison theory (Festinger, 1954) allows athletes to engage in horizontal, upward, and downward peer comparisons for realistic self-evaluation. Guided by learning visualization principles (Brusilovsky et al., 2016), displaying individual and team averages within a unified visual component facilitates immediate, intuitive performance benchmarking. This benchmarking directly supports goal-setting theory (Locke and Latham, 2013) by enabling players to establish self-determined, challenging yet attainable SMART goals (Weinberg and Gould, 2010). Because the developed efficiency norms accurately classify performance, young athletes can ground their short-term, long-term, and competition-specific goals in empirical reality, effectively avoiding discouragement or unreachable performance targets.

To sustain intrinsic motivation, the design leverages cognitive evaluation theory (CET; Deci and Ryan, 1985), assuming that systematic feedback and competence reinforcement directly enhance self-determination. While commercial gamification often functions as a superficial layer, reviews of sports applications indicate that it significantly maximizes long-term user engagement (Tóth and Lógó, 2018). By combining CET with the Fogg behavior model (Fogg, 2021), the application utilizes regular, automated performance notifications as behavioral triggers. This digital iteration modernizes historical non-digital performance tracking, which relied on physical logbooks (Burton and Raedeke, 2008) or sheets (Karageorghis and Terry, 2011) and manual badge distribution (Kazak, 2015). This sport-specific integration marks a distinct shift from educational technology designs like MobilAnalitik (Kokoç and Hayit, 2020), which relied on a self-regulated learning model. While both designs share Festinger's social comparison principles, the mobile app substitutes general learning theories with sport-contextual frameworks (CET and goal setting) to isolate athletic performance variables. Methodologically, this mirrors the action design research (ADR) approach by (Connell and Spencer 2020) in elite football, which demonstrated that implementing a shared, iterative performance analysis system significantly improves communication and technical-tactical understanding between coaches and players.

In line with the user-centered design (UCD) mandate to specify the context of use (Huang et al., 2019), the target audience's digital tendencies were evaluated using the “Digital 2023: Turkey” report (Kemp, 2023). Recognizing that digital natives utilize mobile environments for information-seeking, social networking, and tracking sports figures (Kemp, 2023; Özsari and Saykili, 2020), an interactive, cross-platform Web 2.0 framework compatible with Android and iOS was deemed ideal.

Finally, to establish objective goals, basketball efficiency norms were developed using Erkuş and Selvi's (2019) 10-stage methodology. While previous Turkish sports norming studies focused exclusively on anthropometric and motoric data for early talent selection in athletics (Bayraktar et al., 2010) and wrestling (Bayraktar et al., 2011), this study utilizes live competition statistics. To maximize motivation, norm tiers were assigned positive badges (MVP Candidates, All-Stars, Stars, Average Players, Rotation Players), conceptually inspired by Hollinger's NBA Player Efficiency Rating (PER; Hollinger, 2005). Expert evaluations noted that the ±1 Z-score range generated a wide “Stars” tier; hence, a parallel rank-based percentile matrix was engineered. Naming the 50th percentile and above as “National Player Candidates” directly mirrors real-world talent-selection thresholds (Güllich, 2013). Consequently, although the contribution of our norm development does not constitute groundbreaking mathematical originality, its novelty lies in the adaptive transplantation and recalibration of these professional metrics to a previously under-researched youth basketball context, where structured age- and season-specific distribution benchmarks were lacking.

Competitor analysis against the Web 1.0 TBF App and the scout-centric InStat application revealed that both lack comprehensive goal-setting mechanisms and deliver gamified elements irregularly. This app bridges this technological gap by systematically embedding goal-setting and structured feedback into a single Web 2.0 ecosystem. However, the structural coexistence of discrete components—such as setting up separate goal-input screens alongside peer-comparison metrics—does not propose a fundamentally new psychological mechanism or a transcendent theoretical model that supersedes these independent paradigms. Rather than representing a major theoretical innovation, this architecture should be evaluated as a pragmatic technological synthesis. The system simply operationalizes established theories concurrently within a single digital interface to test their collective feasibility in youth sports engineering. This synthesis was validated through collaborative interviews with software developers and designers, bridging theoretical sports psychology and practical mobile engineering to initiate the development phase.

### 4.2 Developing phase

To operationalize the theoretical models established in the planning phase, the technical development was structured around a scalable, full-stack architecture balancing development efficiency with robust system communication.

For cross-platform rendering, the Flutter framework coupled with the Dart programming language was selected. This hybrid mobile deployment strategy enabled single-codebase development that rendered natively and responsively on both Android and iOS. From a DBR perspective, this selection minimized the significant financial and temporal costs typically associated with separate native application pipelines, thereby optimizing resources for the subsequent empirical evaluation and improvement cycles.

To support dynamic data interactions, the backend architecture used PHP with the Laravel framework, serving as a centralized RESTful API. The implementation of REST API architecture provided a standardized, lightweight communication bridge between the client-side frontend (Flutter) and the server-side relational database (MySQL). This architecture ensured that objective basketball performance metrics and user-generated goal parameters were exchanged and synchronized in real time via standard HTTP. Furthermore, using Laravel's database migration system served as a strict version-control framework, allowing the engineering process to remain highly adaptable to schema changes driven by ongoing stakeholder evaluations.

Methodologically, this infrastructure marks a technological evolution from previous digital intervention designs in educational research, such as MobilAnalitik (Kokoç and Hayit, 2020), which relied on the Apache Cordova framework and standard web technologies (HTML5, CSS3, JavaScript). While the literature demonstrates that technology selection inherently reflects the distinct scope of each intervention, the shift toward modern hybrid engines such as Flutter underscores a growing trend in user-centered sport and educational engineering. Ultimately, this phase demonstrates that integrating rapid, responsive frontend deployment with a decoupled, API-driven backend provides the essential technical stability needed to deliver complex psychological interventions—such as automated performance feedback and individualized goal tracking—to youth athletes in real-world field conditions.

### 4.3 Improving phase

While the literature demonstrates diverse methodological approaches to design optimization—ranging from quantitative questionnaires (Işcan, 2020) and standardized scales (Orhan Göksün et al., 2018) to semi-structured interviews (Kokoç and Hayit, 2020)—this study employed a qualitative usability testing protocol. In user experience (UX) research, qualitative usability verification is a paramount method for evaluating a prototype's operational viability, diagnosing interface friction points, and identifying emergent user needs directly in the field context (Cirucci and Pruchniewska, 2022).

The inductive content analysis of the athletes' testing sessions yielded critical conceptual themes: WEB 2.0 app, user-friendly app, personalized app, customized app, notification, indirect advantages, and suggestions. From a software engineering and human-computer interaction perspective, these empirical themes confirm that this app aligns closely with the functional suitability and usability dimensions outlined in the ISO/IEC 25010 software product quality framework.

Specifically, the user-friendly, customized app themes map directly to the usability sub-characteristics of appropriateness, recognizability, learnability, and operability. The qualitative feedback indicated that the young basketball players could effortlessly navigate the interface, recognize the psychological relevance of their data metrics, and manipulate the system to align with their subjective needs. Furthermore, the notification and personalized app themes satisfy the core criteria of functional appropriateness and completeness. Rather than offering generic, rigid telemetry, the application successfully delivers individualized goal-tracking and automated behavioral triggers (notifications) tailored to the athlete's specific norm stratum. Ultimately, validating the prototype through the subjective lenses of the target audience ensured that the technical architecture was optimized not just for software compliance but also for sustained athlete engagement and behavioral self-regulation.

The mixed methods integrative results showed that the players are divided into five norms; norm development has consistency according to the experts' opinions, and these norm ranges were named “MVP Candidates,” “All-Stars,” “Stars,” “Average Players,” and “Rotation Players,” and utilized as badges in the mobile app design. Furthermore, these results showed the contribution of quantitative and qualitative analyses in the planning phase to the design development phase. Qualitative findings (the opinions and experiences of the youth players) supported the quantitative results and informed the design improvement phase. Furthermore, these results showed that qualitative usability testing, combined with survey questions, enabled extensive data collection on players' experiences and opinions during the improvement phase. Overall, while the findings of the planning phase helped drive the development phase, the findings from both phases helped carry out the improvement phase. The integrated mixed-methods findings also indicated that hybrid applications with user-centered design should be developed for niche groups such as the research sample.

## 5 Conclusion

This research aimed to develop a mobile application using quantitative and qualitative data across three phases (planning the design, developing the design, and improving the design), combining norm development research and user experience research within a design-based research methodology.

**Figure 5:**
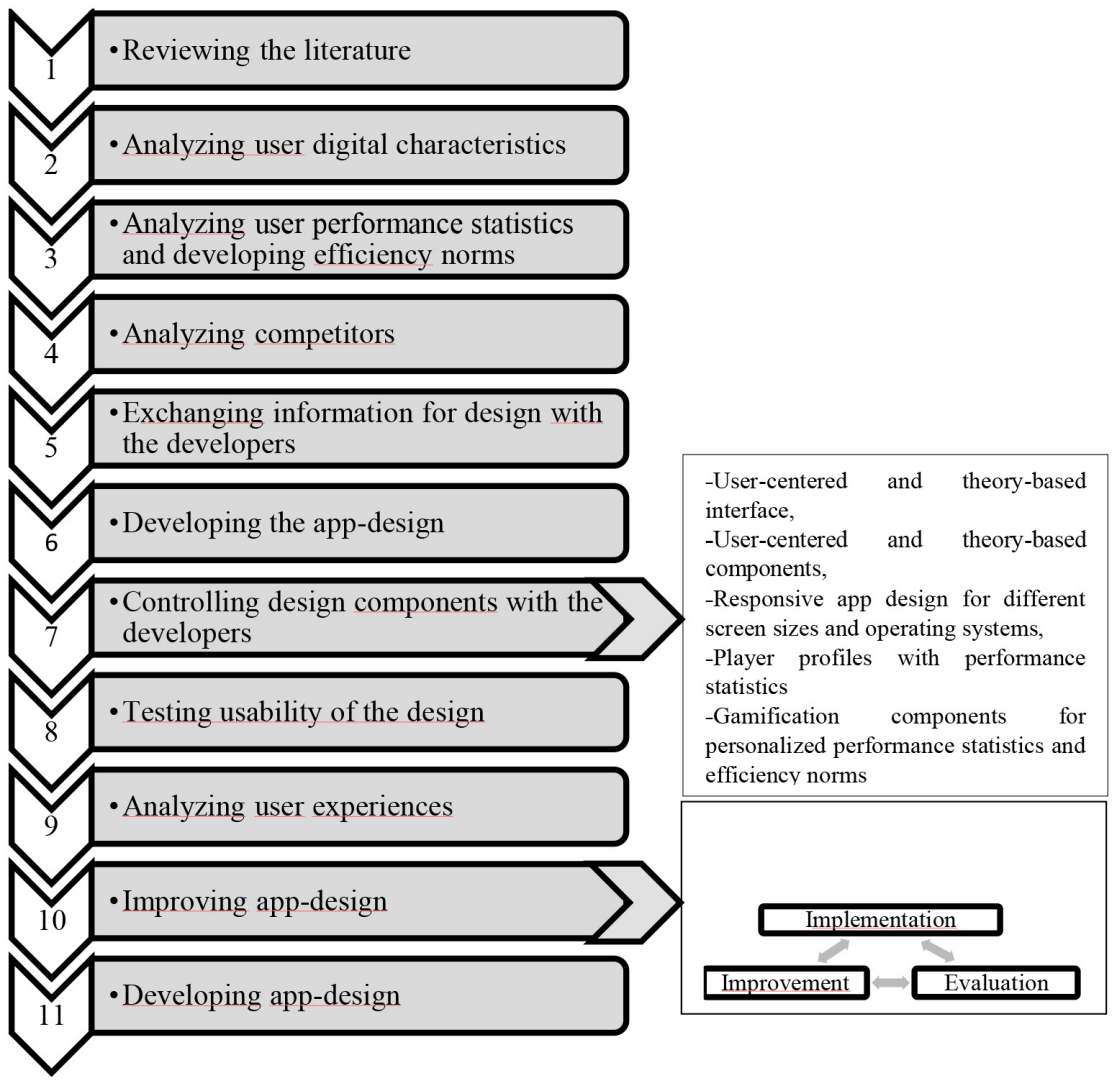
Mobile app development protocol.

Design-based research is a pragmatic research method (Wang and Hannafin, 2005), and it leads to knowledge production that can support both theoretical and practical aspects for designers (Eric et al., 2003). In this context, at the end of this user-centered design-based research, a mobile application called Stats & Goals was developed to improve youth basketball performance (Figure 5). Stats & Goals will help players track match statistics, set individual performance goals, and compare their performance against themselves and their peers. Opportunities of Stats & Goals:

- Tracking performance statistics.
- Players' profiles.
- Personalized players' norms.
- Compare features for players.
- Goal-setting feature for players.
- Gamification.

Design-based research (DBR) leads to knowledge production, including contextually sensitive design principles and theories that can help designers theoretically and practically at the end of the process (Wang and Hannafin, 2005; Eric et al., 2003). In this context, a protocol was developed and proposed for researchers who will develop similar applications arising from this research (Figure 4). Furthermore, this design-based research (DBR) employed a user-centered design (UCD) approach, which has two types of user-centered and learner-centered design frameworks (Huang et al., 2019). Although the DBR methodology is guided by core universal principles, each DBR initiative remains inherently unique, adapting structurally to its specific operational context (Eric et al., 2003). In this context, by integrating these two methodological perspectives, the current study presents a distinct empirical model that bridges behavioral tracking and active user input, thereby contributing to the existing literature as a novel user-centered design-based research framework in youth sports.

### 5.1 Limitations

This research has 4 limitations. First, data from male players across the five seasons of the Turkish Basketball Youth League were used only for norm development. The Turkish Basketball Youth League Girls' Category was not included in the study because it was newly established and not yet mature. Moreover, during these five seasons, some issues that may affect player stats arose. Since Trabzonspor withdrew from the league in the 2018–2019 season, a total of 19 teams competed that season. While the 2019–2020 season was interrupted by the COVID-19 pandemic, the 2020–2021 season was played under a short-season (bubble) format due to pandemic-related measures. To minimize the effect of these differences on norm development, player statistics were divided by the number of matches. Consequently, because the sample is exclusively limited to male athletes, is derived from a single-country dataset (Türkiye), and lacks a longitudinal evaluation of its long-term behavioral effectiveness, the findings have limited generalizability. Therefore, these outcomes should be interpreted with caution within this specific competitive and socio-cultural context, highlighting the need for female basketball players, diverse international youth academies, and longitudinal tracking studies.

Second, TBF's efficiency score was adopted in the research. This efficiency score was assumed to adequately explain player statistics. The efficiency scores used in the literature are limited in their ability to reflect performance in the field. Although some studies suggest separate formulas for offensive and defensive performance, these formulas are not without limitations. Basketball statistics are mostly published as records of players' movements with the ball (points, assists, rebounds, blocks, and turnovers) or of illegal movements (fouls). Consequently, because traditional metrics fail to encapsulate vital tracking data for off-ball defensive indicators (such as help-and-recover, deny-the-ball, active closeouts, and defensive rotations), players with a defense-oriented style systematically exhibit lower efficiency ratings than their offensive peers. In other words, although they contribute a lot to the team's performance and wins, they do not contribute enough to their own individual statistical performance, and they are not as visible as other players. Just as a commonly used formula, such as BMI, classifies muscular individuals as obese, it is also difficult to evaluate the efficiency of players who are good at defense. In other words, it should be noted that formulas that try to evaluate more than one variable in a single score have limitations.

Third, players were selected with nested sampling for their app experiences. So, it is not known whether differences in athletes' opinions affect the app's design. Moreover, while a sample of five users is widely recognized in the human-computer interaction literature as methodologically sufficient for formative software evaluations and for identifying core interface bottlenecks, it is not intended to draw broad, generalized conclusions about definitive user experience patterns or long-term behavioral and motivational effectiveness across the entire league. In addition, the five players evaluated had a higher mean age and operated at a higher athletic performance tier than the all-norm population. Although high-performing and elite youth athletes represent 75% of the sample, their interactions may not fully reflect the experiences of the wider target population due to their distinct psychological coping mechanisms, higher baseline motivation, and differing data-tracking priorities compared to average or rotation players. Therefore, these qualitative outcomes should be interpreted with caution.

Fourth, the researcher sought to collect the players' position data. However, as expected, while some players declared a single position (PG, SG, SF, PF, or C), others declared dual positions (PG-SG, SG-SF, SF-PF, or PF-C). This did not allow for comparisons for these five positions. Therefore, it was decided to collect players' positions when they download the app and enter their personal data. However, this decision prevented the development of norms for player positions.

In other words, the generalizability of the developed app was limited to the context of this research, to players' data from the TBF official website, and to the views and experiences of players and coaches who participated in the research.

### 5.2 Recommendations and future research

Stats & Goals is an application that includes performance, competition, short-term, long-term, and individual goals. However, it is not an application that covers outcome, team, and process goals. Applications that include these features, as well as process, outcome, and team goals, may warrant further research. In terms of process goals, an application with a library of player videos showing players' performance in the match is suggested. In other words, it should have a user-specific video analysis feature like the InStat Basketball Scout App. To push the boundaries of process-oriented athlete tracking, future studies should investigate integrating wearable sensors (e.g., IMUs, heart rate monitors) with AI-driven performance prediction algorithms to deliver real-time, automated feedback. It should not be forgotten that more multidisciplinary researchers and more budget are needed to develop such applications. It should not be forgotten that more multidisciplinary researchers and more budget are needed to develop such applications. For outcome and team goals, it is recommended to identify the features that distinguish better-performing or more successful teams from others and to include components that enable coaches to use the application specifically for teams. Ultimately, evaluating the definitive behavioral efficacy of these complex systems will require rigorous randomized controlled trials (RCTs) alongside longitudinal athlete-monitoring frameworks across multiple competitive seasons.

While developing Stats & Goals, only the young male players' performance statistics were used, and norms and percentages were developed by season and age for them. Therefore, researchers may focus on developing apps that reflect players' norms by position, establishing position-specific performance models in future research. These apps will be useful for learning team roles in team-building literature. In addition, according to social comparison theory, comparisons by position will help players more with setting goals and learning team roles. Furthermore, conducting comprehensive validation of female athletes by creating apps that cover young female players in BYL and all players in other leagues in Türkiye will contribute more to Turkish basketball by having a broader impact.

## Data Availability

The raw data supporting the conclusions of this article will be made available by the authors, without undue reservation.
